# Spinal Cord Injury as a Model of Bone-Muscle Interactions: Therapeutic Implications From *in vitro* and *in vivo* Studies

**DOI:** 10.3389/fendo.2020.00204

**Published:** 2020-04-15

**Authors:** Marco Invernizzi, Alessandro de Sire, Filippo Renò, Carlo Cisari, Letterio Runza, Alessio Baricich, Stefano Carda, Nicola Fusco

**Affiliations:** ^1^Physical and Rehabilitative Medicine, Department of Health Sciences, University of Eastern Piedmont, Novara, Italy; ^2^Rehabilitation Unit, “Mons. L. Novarese” Hospital, Vercelli, Italy; ^3^Innovative Research Laboratory for Wound Healing, Department of Health Sciences, University of Eastern Piedmont, Novara, Italy; ^4^Physical Medicine and Rehabilitation Unit, University Hospital “Maggiore della Carità”, Novara, Italy; ^5^Division of Pathology, Fondazione IRCCS Ca' Granda, Ospedale Maggiore Policlinico, Milan, Italy; ^6^Neuropsychology and Neurorehabilitation Service, Department of Clinical Neuroscience. Lausanne University Hospital (CHUV), Lausanne, Switzerland; ^7^Division of Pathology, IEO - European Institute of Oncology IRCCS, Milan, Italy; ^8^Department of Oncology and Hemato-Oncology, University of Milan, Milan, Italy

**Keywords:** spinal cord injury, bone, muscle, bone loss, osteoporosis, sarcopenia, rehabilitation

## Abstract

Spinal cord injuries (SCIs) represent a variety of conditions related to the damage of the spinal cord with consequent musculoskeletal repercussions. The bone and muscle tissues share several catabolic pathways that lead to variable degrees of disability in SCI patients. In this review article, we provide a comprehensive characterization of the available treatment options targeting the skeleton and the bone in the setting of SCI. Among the pharmacological intervention, bisphosphonates, anti-sclerostin monoclonal antibodies, hydrogen sulfide, parathyroid hormone, and RANKL pathway inhibitors represent valuable options for treating bone alterations. Loss phenomena at the level of the muscle can be counteracted with testosterone, anabolic-androgenic steroids, and selective androgen receptor modulators. Exercise and physical therapy are valuable strategies to increase bone and muscle mass. Nutritional interventions could enhance SCI treatment, particularly in the setting of synergistic and multidisciplinary interventions, but there are no specific guidelines available to date. The development of multidisciplinary recommendations is required for a proper clinical management of SCI patients.

## Introduction

Spinal cord injuries (SCIs) encompass a spectrum of conditions associated with modifications in the function of both central and peripheral nervous systems. Due to the crucial role of the spinal cord in linking the brain with the body, these alterations may lead to dramatic consequences in terms of motor, sensitive, and visceral controls (1).

A complex variety of biological pathways arising in both bone and muscle tissues plays a clinically relevant role in SCIs. Hence, these patients experience important changes in the bones (e.g., osteoporosis) that cause a significantly increased risk of fractures, even after minor traumas (e.g., transferring or sitting) (2, 3). These clinical conditions lead to further immobilization of the patient, increased spasticity, pressure ulcers, and in general worsened disability (3). Bone tissue loss after SCIs starts rapidly and grows in the first 2 years after the injury (4). In chronic SCIs, the skeleton of the lower third of the femur and upper third of the tibia may be subjected to 70% of decreased mineral density (5, 6). Muscle modifications further increase the patients' fragility because they lead to immobilization, increased fracture risk, pressure sores, thrombosis, overpressure, chronic pain, and psychosocial issues (7). The loss of mass in the muscles below the SCI is remarkable, reaching up to 40% in the first 2 years after the injury (8). Regrettably, despite muscle atrophy is macroscopically more evident than osteoporosis, this phenomenon is often underestimated (7).

In this scenario, understanding the biological interplay of the bone and muscle tissues is crucial for proper clinical management of SCIs. Here, we sought to provide a comprehensive portrait of the potentials and limitations of the various treatment options available (or proposed) to date for both osteoporosis and muscle atrophy occurring after SCIs.

## Pharmacological Approaches to Bone Alterations

The use of single, combination, or sequential therapy protocols in the management of bone alterations in SCI is a matter of controversy. After the achievement of clinical benefits, the discontinuation of osteoanabolic treatments could result in a rapid loss of the newly gained bone (9–12). For this reason, clinicians are recommended to promptly start other anti-resorptive therapies after osteoanabolic interventions discontinuation. At present, there are no widely adopted guidelines about the most reliable pharmacological strategy.

### Bisphosphonates

Bisphosphonates are the most used class of drugs in the prevention and treatment of osteoporosis (13). Several studies have assessed the efficacy of these anti-resorptive drugs in acute and chronic SCIs. A meta-analysis provided circumstantial evidence to suggest that the early administration of bisphosphonates can reduce SCI-related osteoporosis (13). It should be taken into account, however, that this hypothesis is biased by the small sample size of the few clinical trials available to date (14). Moreover, despite many groups showed that bisphosphonates may be particularly effective at the hip level, only a few information is currently available on their role at the distal third of the femur and/or at the proximal third of the tibia. A recent, non-randomized study on the yearly administration of zoledronic acid failed to show an improvement in the bone mass density (BMD) at this level, being associated, on the contrary, with a reduction in bone mass (15). Therefore, several doubts exist about the efficacy and the safety profile of bisphosphonates in patients with SCI. Due to these issues, at the present time, prophylaxis of osteoporosis with bisphosphonates in SCI remains an interesting area of research but it is not yet ready for prime time (16). On the other hand, alendronate showed significant results in maintaining or even increase BMD after previous osteoanabolic interventions (9, 10, 17). These observations suggest that a sequential therapy scheme with alendronate after teriparatide treatment is likely to prevent bone loss, increase bone mass, and preserve bone strength at the spine and hip in SCI patients.

### Anti-sclerostin Antibodies

Murine SCI models showed that anti-sclerostin antibodies could preserve the osteocyte morphology and structure, blocking the skeletal deterioration after either motor-incomplete (18) or motor-complete SCI (15). A recent study assessed the efficacy of these antibodies in reversing the bone loss in a rodent model after motor-complete SCI (19). Animals treated with 25 mg/kg/week for 8 weeks (starting at 12 weeks after SCI) showed a significant increase in BMD, structure, and mechanical strength. Anti-sclerostin antibodies acted in the case group through osteoclastogenesis inhibition and concomitant osteoblastogenesis stimulation. Untreated SCI mice showed a significant reduction in BMD and the deterioration of bone structure at the distal femoral metaphysis. These data suggest that sclerostin antagonism might be a viable therapeutic option not only to prevent bone loss after acute SCI but also in the chronic setting (19).

### Hydrogen Sulfide

Hydrogen sulfide (H2S) is a colorless and poisonous gas acting as a gas transmitter to regulate different signaling pathways (20). It has been recently hypothesized that abnormal H2S metabolism could be linked to defects in bone homeostasis (21). Treatment with an H2S donor (GYY4137) increase bone formation, preventing the loss of trabecular bone in case of estrogen deficiency (21). Whether H2S might have anti-osteoporosis effectiveness in motor-complete SCI models has recently been investigated (22). Rats were treated with an intraperitoneal injection of 0.1 ml/kg/day of 0.28 mol/l NaHS (a donor of H2S) for 2 weeks. They showed an increase of femoral and tibial BMD, bone volume fraction (bone volume/total, BV/TV), trabecular thickness and number, and a concomitant reduction of trabecular separation in proximal tibiae. Furthermore, rats treated with NaHS showed an increased mineral apposition rate (MAR), bone formation rate (BFR), and osteoblast proliferation (22). Given that an increased ROS production is one of the main mechanisms related to bone and muscle modifications after SCI, all these results suggest a potential role of H2S treatment in SCI-related bone loss.

### Parathyroid Hormone

Parathyroid hormone (PTH) is a well-known player in calcium metabolism. The subcutaneous intermittent administration of PTH has been used for two decades as a valid anabolic therapeutic agent for osteoporosis treatment (23, 24). A recent study investigated whether PTH administration might reduce bone loss in SCI female mice (25). PTH (80 mg/kg) or vehicle was injected subcutaneously daily for 35 days. Isolated tibias and femurs were assessed through microcomputed tomography scanning (micro-CT), histology, and serum bone turnover markers. SCI-vehicle animals reported a 49% reduction in fractional trabecular bone volume and 18% in trabecular thickness compared to sham-vehicle controls. Moreover, the authors found 15% lower femoral cortical thickness and 16% higher cortical porosity in SCI-vehicle animals compared to sham ones. PTH treatment restored 78% of bone volume in SCI animals. In addition, histomorphometry showed a marked decline in osteoblast and osteoclast number and a 35% reduction in bone formation rate. On the other hand, in SCI-PTH animals the number of osteoblasts and osteoclasts was preserved, and the bone formation rate was enhanced. These results suggest a potential therapeutic role of subcutaneous intermittent PTH treatment in acute SCI patients, however to date, data in humans are lacking. Recently, abaloparatide, a parathyroid hormone-related protein (PTHrP), was approved by Food and Drug Administration (FDA) for the treatment of severe post-menopausal osteoporosis (26). A recent study performed by Sahbani et al. compared abaloparatide to teriparatide on bone structure, turnover, and levels of RANKL and OPG on wild-type female mice (27). The authors showed that 20–80 μg/kg/day of abaloparatide for 30 days resulted in a significant increase (13%) in trabecular bone thickness and lowered the RANKL/OPG ratio. In light of these results, abaloparatide could be a promising therapeutic agent to counter osteoporosis, and future studies could investigate its efficacy also in SCI patients.

### Denosumab

The receptor activator of nuclear factor-kB ligand (RANKL)/osteoprotegerin (OPG) system is one of the main regulators of bone remodeling (28). Denosumab, a RANKL pathway inhibitor monoclonal antibody, has been widely used in the treatment of osteoporosis (29–33). Increased osteoblast expression of RANKL has been previously observed in unloading conditions, such as SCI, resulting in an increased RANKL/OPG ratio (34–38). These observations have suggested a potential therapeutic role of denosumab in SCI-induced bone loss. Only one work investigated the effects of denosumab on acute SCI in humans, with promising results (33). Compared to RANKLS-positive patients with detectable RANKL, those with undetectable RANKL levels showed better responses both in terms of femoral BMD increase and bone reabsorption markers decrease. Therefore, denosumab has been regarded as a drug potentially able to prevent SCI-related bone loss (38).

## Pharmacological Approaches to Muscle Alterations

To date, there is no robust evidence about local pharmacological agents that can be used to counteract the muscle loss after SCI. Most data currently available are derived from studies on healthy subjects or patients with chronic conditions of which some (e.g., cachexia and wasting) share some pathophysiological pathways with SCI. In this setting, androgens have been extensively studied, either in animal models and in humans. Particularly, testosterone and anabolic-androgenic steroids (AAS) such as nandrolone, as well as the selective androgen receptor modulators (SARMs), have been proposed as pharmacological strategies to hinder the loss phenomena at the musculoskeletal level in different chronic illnesses, such as immunodeficiency, long term glucocorticoid treatment, and aging (39, 40). Data about safety and efficacy on the lean mass increase are available in healthy subjects, aged women, and men and in cancer-related cachexia (40–42). The main factor supporting the use of SARMs instead of testosterone is the tissue selectivity and good tolerability with negligible adverse events. The evidence emerging from cancer cachexia patients suggests a theoretical use of SARMs also in SCI-induced atrophy. The observation that a substantial proportion of male and female individuals experience hypogonadism after acute or chronic SCI represents the rationale of using testosterone and AAS in these patients (43, 44). Several studies have shown a significant effect of testosterone and nandrolone against muscle atrophy in rodents (45, 46). These results have been partially replicated in a small trial on SCI patients, in which the Authors showed that the correction of hypogonadism by 12 months of testosterone therapy can increase lean tissue mass for the whole body, leg, and arm (47). A recent randomized clinical trial showed that the combination of electrical stimulation exercise training and testosterone replacement therapy can increase muscle mass in the lower extremities of SCI patients (48). This effect was not present in patients only treated with testosterone without exercise.

## Exercise and Physical Therapy

Exercise is the first treatment to increase muscle mass and there is growing evidence that the basic mechanisms that regulate muscle hypertrophy can be used to counteract disuse atrophy (40). The mechanisms by which physical exercise induce muscle hypertrophy have not been completely elucidated. The IGF-1/PIP3K/Akt pathway, even if it is considered a key mediator of normal muscle development, is not able to explain alone the exercise-induced muscle hypertrophy (49). On the other hand, mTORC1 appears to be crucial in load-induced muscle growth (50), as phospholipase D and phosphatidic acid that active mTORC1 (51). In a rodent model, exercise can protect the skeletal muscle against oxidative stress and proteolysis (52). Eccentric contractions can increase phosphatidic acid levels and induce muscle growth on mice, confirming the importance of this mechanism (53).

### Physical Exercise and Treadmill

A recent study performed on 42 male Wistar rats showed that stretching is helpful for the correction of contractures after SCI (54). In addition, the Authors observed that heat is more beneficial than cold to increase the effectiveness of stretching in terms of restoring range of motion and decreasing muscle contractures. Lately, it has been demonstrated that 9-week treadmill training in mice affected by incomplete SCI is effective in preventing atrophy of fast-twitch muscles, with limited effects on slow-twitch muscles and muscle fiber type composition of medial gastrocnemius, soleus, and tibialis anterior (55). In healthy humans, several studies have shown that eccentric training (i.e., motion of a muscle while it is lengthening under load) can increase muscle mass and muscle strength more than concentric exercise [i.e., shortening of a muscle while it is contracting; (56)]. Exercise can also reduce the expression and production of myostatin (57–59).

### Functional Electrical Stimulation Rowing Exercise

In the early 1990s, functional electrical stimulation (FES) rowing exercise has been developed as an aerobic training program for SCI patients (60, 61). This approach consists of coordinated voluntary upper body exercise combined with electrical stimulation of the leg large muscle groups to produce a near-full-body exercise. Recently Lambach et al. (62) performed a study on four adults with recent (<2 years) traumatic, motor complete SCI, undergoing a 90-session FES rowing exercise program, and attending 30-min FES training sessions for 3 times/week. They showed that trabecular BMD in the femur and tibia decreased for all participants after 30 sessions, with a rate of loss slowed or reversed between the 30th and 60th sessions, with the little-to-no bone loss for most participants during the period intercurrent from 60th and 90th sessions. However, further studies with larger cohorts of participants are needed to confirm the observation of FES rowing exercise in SCI individuals.

### Whole-Body Vibration

In the recent past, whole-body vibration (WBV) effects on bone architecture after SCI have been investigated with inconsistent results. In 2016, Dudley-Javoroski et al. showed that 12 months of vibration training did not preserve BMD or trabecular architecture in subjects affected by chronic SCI, showing bone tissue insensitivity to mechanical loading interventions (63). These results are not in line with the previous studies performed on rats, where WBV seemed to attenuate the bone loss that commonly occurs during the early acute stages after SCI (64). On the other hand, WBV seems to have a positive role in ankle spasticity, balance, and walking ability in patients with incomplete SCI at the cervical level (65), suggesting a potential synergistic role with other therapeutic interventions. Only a handful of studies investigated the use of WBV to improve muscle function in patients with SCI (66). Among them, a single study evaluated functional parameters such as walking speed (67), whereas the others took into account only evaluated electrophysiological data. One study failed to show any modifications in muscle cross-sectional area or muscle density after 40 weeks of treatment (68). It should be noted that available studies have been conducted on small sample sizes, with heterogeneous protocols (intensity, duration, frequencies). Therefore, no strong conclusions can be drawn at present time.

### Epidural Electrical Stimulation

**Figure 1:**
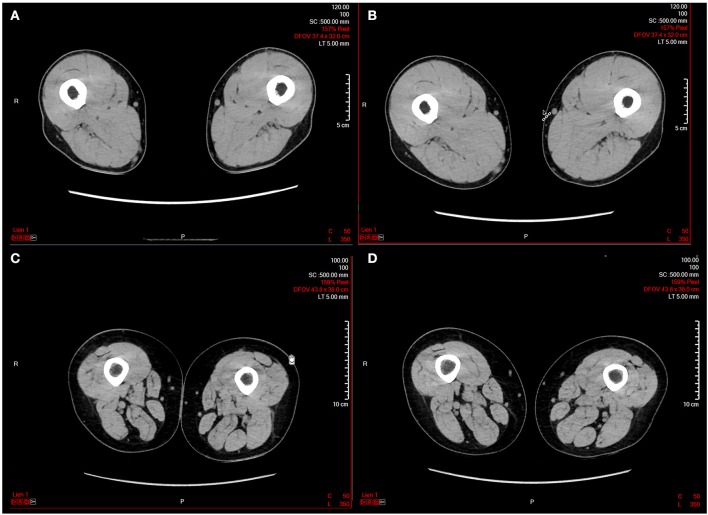
Representative computed tomography (CT) scans before and after a 16 weeks epidural electrical stimulation (EES) protocol in two patients with spinal cord injury. **(A)** Patient 1 before EES; **(B)** patient 1 after EES; **(C)** patient 2 before EES; **(D)** patient 2 after EES. The images have been kindly provided by Prof. G. Courtine and J. Bloch.

Epidural electrical stimulation (ESS) is a promising technique for improving motor recovery in chronic patients with SCI (69, 70). Preliminary results showed that the application of ESS improving both motor control and muscle mass in the lower extremity of patients with severe paralysis or plegia (Figure 1). This effect could be ascribed to the stimulation of the posterior roots, enabling muscle contraction (69). This mechanism is different from that obtained by FES since muscle contraction is mainly indirect. Interestingly, spasticity, which is another indirect, reflex activity in patients with SCI, is associated with partially preserved muscle mass (70).

## Nutritional Interventions

Despite a healthy diet is recommended in SCI individuals, there is a substantial lack of nutritional guidelines for these patients (71–73). In addition, their altered body composition limits the accurate measurement of the energy intake and the need (74, 75). A recent systematic review and meta-analysis investigated the nutritional status of adult subjects with chronic SCI (76, 77). The authors showed that mean total energy (1,876 kcal/day), fiber intake (17 g/day), vitamins A, B5, B7, B9, D, E, potassium, and calcium were below the levels recommended by the United States Department of Agriculture (USDA). On the other hand, protein (319 kcal/day) and carbohydrate (969 kcal/day) intake and vitamins B1, B2, B3, B12, C, K, sodium, phosphorus, copper, and zinc exceeded the USDA recommendation. Of note, the fat intake (663 kcal/day), vitamin B6, iron, and magnesium were within limits (77). Supplementation with creatine to improve muscle strength in patients with chronic SCI has been also evaluated, with conflicting results. Two studies found no effect of creatine supplementation either on hand strength (78) or wheelchair performance (79), while two studies found some positive effects on upper extremity strength and function (80, 81). All these diverse correlations highlight the need for a prompt and precise nutritional assessment in SCI patients, focusing on correct daily energy intake and a higher fiber and micronutrient intake (i.e., vitamin D and calcium). Future studies are required to investigate not only the nutritional health status but also the adequate nutritional supplementation and its synergism with other interventions in SCI patients.

## Synergistic and Multidisciplinary Interventions

There is no unequivocal evidence about the superiority of a single therapeutic intervention to counteract the modifications in bone and muscle tissues after SCI. Given the complex and multifaceted nature of these conditions, it is conceivable that the best therapeutic strategy consists of the synergistic use of several different interventions in order to optimize the outcome at bone and muscle level in SCI patients.

### Zoledronic Acid Plus FES Rowing Exercise

Considering the promising role of FES rowing exercise in promoting muscle contraction with combined mechanical loading of lower limb long bones in SCI patients, a therapeutic synergism with drugs acting at the bone level has been proposed (82). This combined strategy could induce both bone resorption inhibition and bone neoformation stimulation. A randomized clinical trial comparing FES-rowing alone with FES-rowing plus zoledronic acid (a powerful bone anti-resorptive drug), have been carried out to investigate the impact of bone health in SCI (82). The latter group had significantly greater cortical bone volume (CBV) and cortical thickness index (CTI) at both distal femoral and proximal tibial metaphyses, compared to the FES-rowing-only group. In the rowing-only group of patients, the cortical compressive strength index (CSI) at the tibial metaphysis showed significant variations based on the amount of exercise performed. These data showed that a combined antiresorptive and FES rowing exercise therapy might improve bone quality and geometry, suggesting that multimodal therapeutic interventions could be beneficial in counteracting the bone and muscle modifications.

### Teriparatide and Whole-Body Vibration

Teriparatide, a 34 amino acid peptide representing the N-terminal bioactive portion of human PTH, is the only anabolic agent approved to enhance bone mass and prevent fragility fractures (83, 84). A recent randomized, double-blind, multi-center, parallel-group clinical trial on 61 individuals with chronic SCI and low bone mass showed that patients treated with teriparatide 20 μg/day showed a significant increase in areal BMD (aBMD) at the spine level after the first 12 months in both groups treated (4.8–5.5%) regardless of the simultaneous administration of WBV or sham WBV (85). This observation is consistent with a marked response in serum markers of bone metabolism. On the other hand, the magnitude of the objective response rate was not significantly related to the treatment strategy. Furthermore, patients treated by teriparatide in the 12-month extension study showed further improvement in terms of spine aBMD (total increase from baseline 7.1–14.4%), which was higher in those initially randomized to teriparatide. Taken together, teriparatide administration is related to an increased spine aBMD skeletal activity and its activity is not increased by vibration stimulation in chronic SCI individuals.

## Future Perspectives

**Figure 2:**
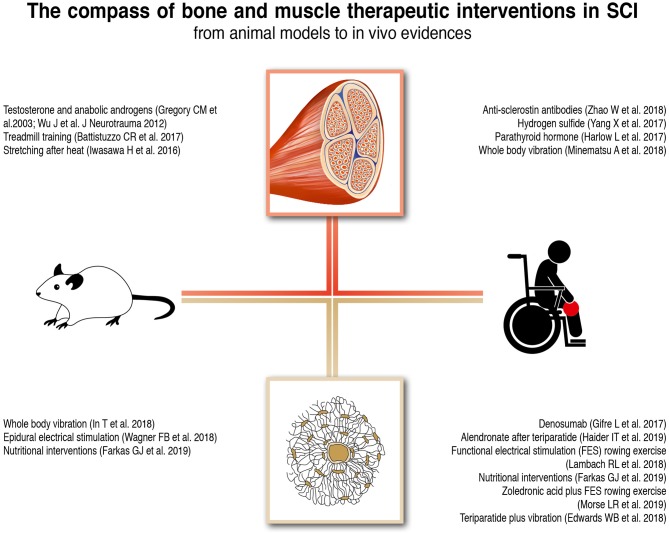
Bone and Muscle therapeutic interventions in SCI animal models and human subjects.

Despite the progress that has been made in the field of regenerative spinal cord medicine, the majority of SCI patients continue to experience not only the direct neurological damage but also the clinically relevant secondary complications affecting the muscle and bone tissues. Due to the scarcity of available data, attention should be paid in making strong statements or recommendations for the management of these patients. However, it is anyway possible to translate some of the existing information into real-life clinical practice and to outline the future directions for primary research and clinical studies. The main bone and muscle therapeutic interventions in SCI animal models and human subjects are outlined in (Figure 2).

Given that the bone and muscle tissues share not only their origin from the mesenchymal multipotent stem cell but also important signaling pathways, response to mechanical stimulation, and complications, it is reasonable to conceive that treatments should ideally target both tissues. From this perspective, physical exercise, nutrition, and electrical stimulation seem to be the most promising and rational way of treating both muscle atrophy and osteoporosis. In particular, the epidural electrical stimulation has the interesting potential of fostering both the neurological recovery and the increase of the muscle mass. Hormonal treatments represent another valuable option but their safety profile needs to be investigated in the long term.

Another future therapeutic option might be the modulation of microRNAs (miRNAs) which are non-coding RNA molecules involved in transcriptional regulation that target pathways of human diseases. They might be considered not only a target to prevent bone and muscle modifications but also a potential powerful therapeutic intervention in improving the functional recovery after SCI. In this regard, it has been recently showed how miRNA-411 and miRNA-340-5p increase could reduce apoptosis, gliosis and glial scar formation, and all the inflammators in SCI rats (86, 87).

## Conclusions

SCIs encompass a wide variety of abnormal conditions that reverberate at the musculoskeletal level. Many secondary modifications show similarities and shared pathophysiological patterns to both muscle and bone tissues. The limited evidences in the literature summarized in this review suggests the need of an early start of any therapeutic intervention. However, clear evidence about the most effective treatment to counter bone and muscle modifications after SCI is missing.

A deeper understanding of the bone and muscle interactions would improve the outcome of the patients affected by several conditions related to musculoskeletal changes, such as SCI, aging, space travels, and prolonged immobility. Additional insights in bone and muscle interactions will be germane for the development of multitargeted specific therapeutic and preventive strategies acting on both bone and muscle tissue. The development of multidisciplinary recommendations is required for the clinical management of these patients at an individualized level.

## Author Contributions

MI and SC designed the study. MI, AS, SC, AB, FR, and LR reviewed the literature. MI, AS, NF, SC wrote the manuscript. All authors revised the paper and approved the final edition.

### Conflict of Interest

The reviewer LP declared a shared affiliation, with no collaboration with one of the authors NF, to the handling editor at time of review. The remaining authors declare that the research was conducted in the absence of any commercial or financial relationships that could be construed as a potential conflict of interest.
